# First person – Krittika Sudhakar

**DOI:** 10.1242/bio.059500

**Published:** 2022-07-25

## Abstract

First Person is a series of interviews with the first authors of a selection of papers published in Biology Open, helping early-career researchers promote themselves alongside their papers. Krittika Sudhakar is first author on ‘Alterations in lifespan and sleep:wake duration under selective monochromes of visible light in *Drosophila melanogaster*’, published in BiO. Krittika conducted the research described in this article while a PhD student (DST-INSPIRE-senior research fellow) in Dr. Pankaj Yadav's lab at SASTRA University, Tirumalaisamudram, India. She is now a Postdoctoral Fellow in the lab of Dr. Adelheid Lempradl at the Van Andel Institute, Michigan, USA, investigating Deciphering the short and long-term effects of nutrition influences *Drosophila* physiology, aging and metabolism.


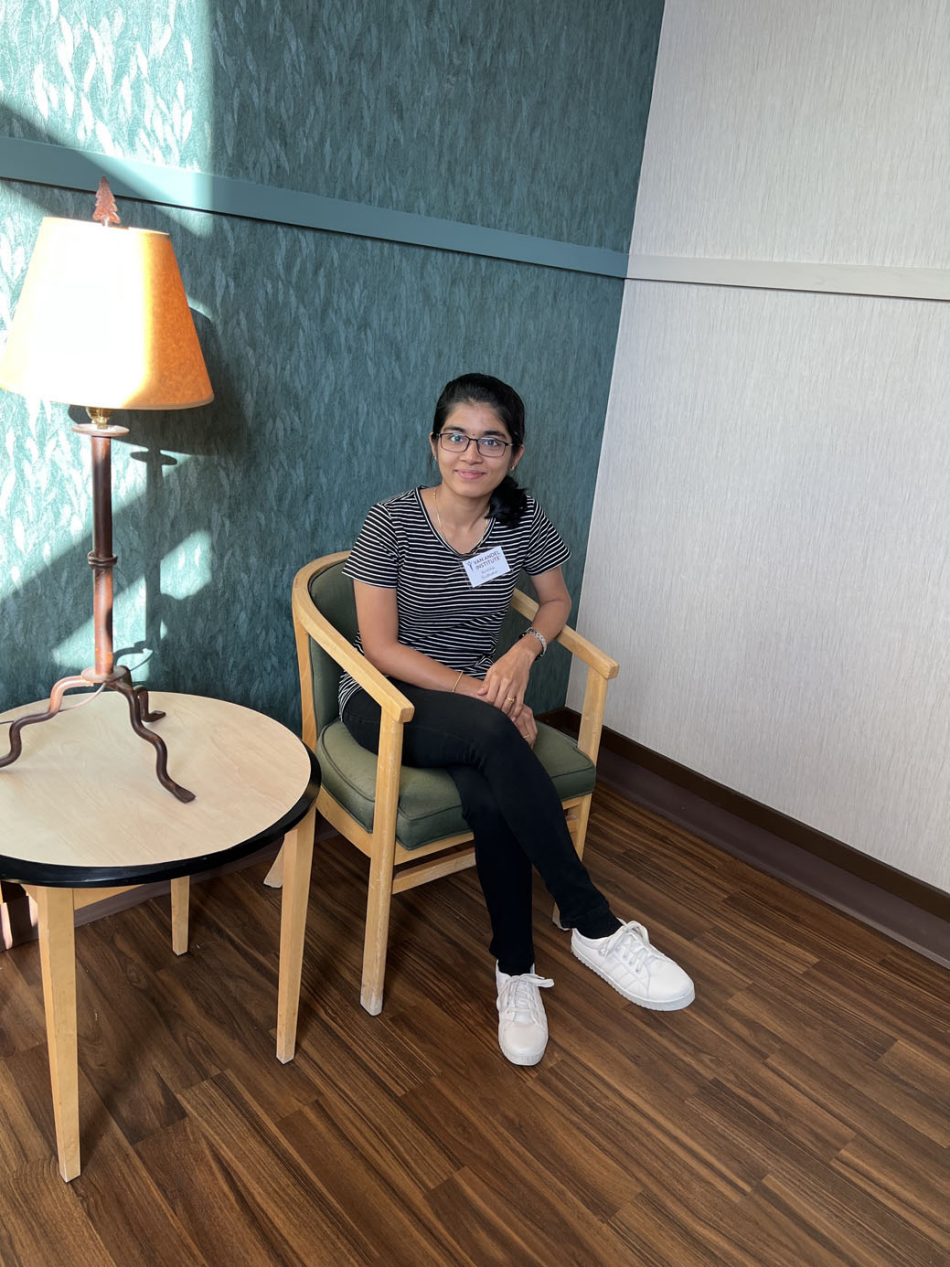


**Krittika Sudhakar**

**What is your scientific background and the general focus of your lab?**

My scientific background is nutrition and aging of fruit flies, *Drosophila melanogaster*. The research of the current article was carried out at Fly Lab (SASTRA University) during my PhD tenure there, wherein we aimed to understand the nutrition, physiology, behavior and the chronobiology of *D. melanogaster*.

“Sharing and explaining my research to non-scientific people is my favorite part of my research career.”

**How would you explain the main findings of your paper to non-scientific family and friends?**

Sharing and explaining my research to non-scientific people is my favorite part of my research career. You can always find me sharing the same to my family and friends. I have told them and would tell them: “We are currently exposed to too much of the digitalized world and gadgets, which increases our proximity to a single-colored light, like blue light, which is emitted from mobile phones, television, computers, etc. This might lead to harmful effects on vision, sleep and wake cycles, etc., and thereby indirectly on our health. We use the lab-bred version of model organism of fruit flies (that one seen on banana in our household) to study the same single colored light effect on their lifespan and sleep:wake cycles. We found that blue and green colored light exposure affects sleep quality and the flies avoided staying in those colors. This eventually cost the flies their lives which decreased their lifespan under violet, blue, green lights. That is our finding here!”

“We found that blue and green colored light exposure affects sleep quality and the flies avoided staying in those colors.”

**What are the potential implications of these results for your field of research?**

These findings will help us understand the sleep:wake cycles of flies under monochromatic lights and for the first time reveal insights on the anticipation index of the flies for a specific phase of the day.

**What has surprised you the most while conducting your research?**

The awe and surprise that I had in my research was to see the little flies, when I load them into their 5 mm activity tubes every time. It never fails to excite me when I see them walking in those tubes with their tiny legs and then exploring their new environment immediately once I transfer. Apart from the unexpected results in my research, this is the best part!

**Figure:**
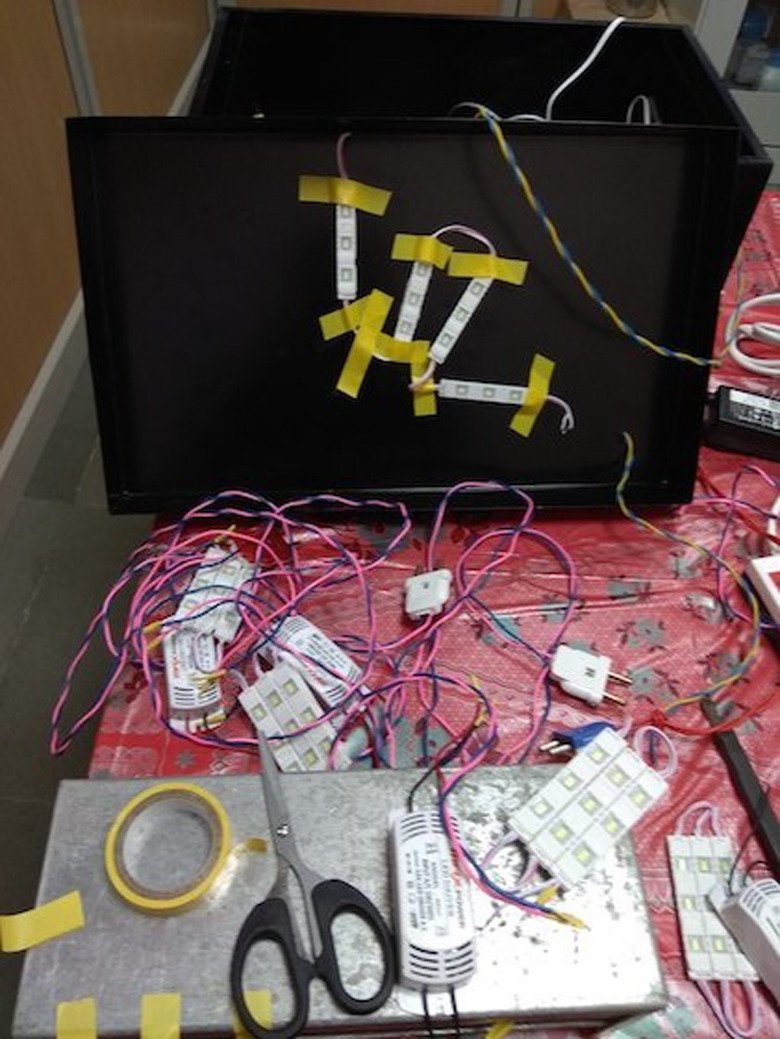
The unusual haywire electrical work for the current biological experiment – biologists can do anything!

**What, in your opinion, are some of the greatest achievements in your field and how has this influenced your research?**

The greatest so far was to identify that blue light had toxic effect on a small invertebrate like fruit flies and to think of its scary implication on humans. Some of the highlights in this field were understanding that fruit flies prefer different colors at different times of the day, and this influenced me to address their anticipation index in my research paper.

**What changes do you think could improve the professional lives of early-career scientists?**

The First Person interview is one of its kind for the exposure it is giving to early-career scientists. I believe that funding and more recognition of their work can improve the mental and thereby the professional lives of early-career scientists.

**What's next for you?**

I am currently standing on my next step, which I have recently climbed from the step where I conducted my PhD research. I have very recently joined as a postdoc with Dr. Adelheid Lempradl's Lab in the Department of Metabolism and Nutritional Programming at the Van Andel Institute, which is a non-profit biomedical research organization. Our lab focuses on *Drosophila* nutrition and metabolism and its inter-generational inheritance. I'm looking forward to this new journey!

**Was this work a part of your PhD work and what do you do at Van Andel Institute currently?**

No, this work was not a part of my PhD thesis, it was another research area we had started during my PhD tenure there. Currently, as a postdoc at the Lempradl's Lab, we try to understand the inter-generational inheritance and the role of maternal nutrition in influencing certain fitness traits, early embryo metabolites and obesity. The work environment and the people here are amazing, and the institute provides great guidance and opportunities to develop one's career. I am greatly enjoying the time and learning new things almost every day!
